# Validation of a Liquid Chromatography-Tandem Mass Spectrometric Assay for Quantitative Analysis of Lenvatinib in Human Plasma

**DOI:** 10.1155/2017/2341876

**Published:** 2017-06-07

**Authors:** Tomoko Ogawa-Morita, Yoshiyuki Sano, Tomoka Okano, Hirofumi Fujii, Makoto Tahara, Masakazu Yamaguchi, Hironobu Minami

**Affiliations:** ^1^Division of Pharmacy, National Cancer Center Hospital East, Chiba, Japan; ^2^Division of Functional Imaging, National Cancer Center, Chiba, Japan; ^3^Division of Head and Neck Oncology, National Cancer Center Hospital East, Chiba, Japan; ^4^Division of Medical Oncology/Hematology, Department of Medicine, Kobe University Hospital and Graduate School of Medicine, Hyogo, Japan

## Abstract

Toward conducting clinical pharmacokinetic studies of an antineoplastic agent, lenvatinib, we developed a liquid chromatography-tandem mass spectrometric assay for its quantitative analysis in human plasma. Analyte (lenvatinib) and internal standard (IS, propranolol) in the plasma were extracted by using acetonitrile and chromatographically separated by using a XTerra MS C18 column with 0.2 mL/min flow and mobile phase starting with 0.1% formic acid in water, followed by increasing percentage of acetonitrile. Detection was performed by using combined reversed-phase liquid chromatography-tandem mass spectrometry (LC/MS-MS) with positive ion electrospray ionization. MS-MS ion transitions used were 427.602>371.000 for lenvatinib and 260.064>116.005 for IS. This study was validated for accuracy, precision, linearity, range, selectivity, lower limit of quantification, recovery, and matrix effect according to the Guideline on Bioanalytical Method Validation in Pharmaceutical Development in Japan. Calibration curve was plotted by using lenvatinib concentrations ranging within 9.6–200 ng/mL, and correlation coefficients (*r*^2^) were in excess of 0.997. Intra- and interday accuracy ranged within 95.8–108.3% with mean recoveries of 66.8% for lenvatinib, and precision was <6.7% at all quality control concentration levels. Matrix effect analysis showed extraction efficiency of 15.7% for lenvatinib. Collectively, these findings demonstrate the feasibility of this method to evaluate kinetic disposition of lenvatinib.

## 1. Introduction

Lenvatinib is an oral inhibitor of multiple receptor tyrosine kinases targeting vascular endothelial growth factor (VEGF) receptors 1–3, fibroblast growth factor (FGF) receptors 1–4, platelet-derived growth factor (PDGF) receptor *α*, ret protooncogene, and v-kit. Lower limit of quantification chemotherapy is well-established and approved for treatment of thyroid cancer [1–4].

Two studies on development and validation of the quantification method of lenvatinib by liquid chromatography-tandem mass spectrometry (LC-MS/MS) have been reported previously [5, 6]. These studies used ER-227326 (IUPAC: 4-{3-chloro-4-[(propylcarbamoyl)amino]phenoxy}-7-methoxyquinoline-6-carboxamide) monomethanesulfonate, which is synthesized by Eisai Inc. as an internal standard. However, it is not commercially available and the synthesis of its stable isotope is expensive. In this study, we developed and validated a liquid chromatography-tandem mass spectrometric assay for quantitative analysis of lenvatinib in human plasma by using propranolol as an internal standard. Propranolol was chosen as an internal standard for lenvatinib because it is more cost-effective than stable isotope, and it causes minimal ion suppression and enhancement effects on the analyte.

We examined selectivity, lower limit of quantification, calibration curve, accuracy, precision, matrix effect, carry-over, dilution integrity, and stability and validated new bioanalytical method for quantification of lenvatinib according to the Guideline on Bioanalytical Method Validation in Pharmaceutical Development in Japan [7].

## 2. Material and Methods

### 2.1. Chemicals and Reagents

Lenvatinib was purchased from AdooQ BioScience LLC (Irvine, CA, USA). Internal standard propranolol (C_16_H_21_NO_2_) and acetonitrile (CH_3_CN, LC-MS/MS hypergrade) were purchased from Merck, Ltd. (Darmstadt, Germany). Methanol (CH_3_OH, LC-MS/MS hypergrade) and formic acid (HCOOH, LC-MS/MS hypergrade) were obtained from Wako Pure Chemical Industries, Ltd. (Osaka, Japan). High-purity deionized water was obtained from an Elix 10-Milli-Q Plus water purification system (Millipore, Ltd., Eschborn, Germany).

### 2.2. Instrumentation

Analyses were carried out by using the Prominence LC-20AB/SPD-20A liquid chromatography system that consisted of a LC-20AB binary pump equipped with an online degasser and an autosampler (Shimadzu Co., Kyoto, Japan). The chromatographic system was operated by using the Analyst software 1.5.1 (AB Sciex, Ltd., Framingham, MA, USA). Separations were conducted by using a 50 mm × 2.1 mm I.D. XTerra MS C18 column (Waters Co., Milford, MA, USA).

Detection was performed with an API3200 quadrupole mass spectrometry (AB Sciex, Ltd., Framingham, MA, USA), equipped with turbo ion spray interface, operated in the positive mode, and configured in multiple reaction monitoring mode. The mass spectrometry system was operated by using the Analyst software 1.5.1 (AB Sciex, Ltd., Framingham, MA, USA).

### 2.3. Liquid Chromatography

Chromatography was performed by using LiChrosolv® LC-MS grade acetonitrile, high-purity deionized water, and formic acid. The mobile phase used for chromatography was composed of two solutions: ultrapure water and 0.1% formic acid (solution A), as well as acetonitrile and 0.1% formic acid (solution B). The flow rate of the mobile phase was set at 0.2 mL/min by using the following gradient elution program: 0% acetonitrile at 0 min, 95% acetonitrile at 10-11 min, 5% acetonitrile at 12 min, and a reequilibration step to the initial solvent from 12 to 15 min.

### 2.4. Mass Spectrometry

The instrument source parameters of mass spectrometry were optimized by performing direct injection and flow injection analysis: curtain gas (CUR): 10; collision gas (CAD): 8; ion spray voltage (IS): 5500; temperature (TEM): 700; ion source gas 1 (GS1): 50; ion source gas 2 (GS2): 80. The singly charged precursor ions for lenvatinib at *m*/*z* 427.603 and propranolol (IS) at *m*/*z* 260.065 were selected in Q1, and each singly charged transition was monitored in Q3: lenvatinib at 371.000 and propranolol at 116.100.

### 2.5. Preparation of Samples

The calibration standard (CS) samples and quality control (QC) samples were independently prepared. To prepare 50 *μ*g/mL stock solutions, approximately 0.2 mg lenvatinib was exactly weighed and dissolved in 3.3 mM HCl/CH_3_OH. Stock solutions were diluted with 3.3 mM HCl to obtain CS and QC working solutions. Subsequently, CS and QC samples in plasma were prepared by diluting the corresponding working solutions with drug-free human sodium heparinized plasma. CS samples and QC samples were prepared to reach the final concentrations of CS samples at 200 ng/mL, 145 ng/mL, 96 ng/mL, 48.3 ng/mL, 19.3 ng/mL, and 9.6 ng/mL and the final concentrations of QC samples at QC-LLOQ: 9.6 ng/mL, QC-low: 19.3 ng/mL, QC-mid: 96 ng/mL, and QC-high: 150 ng/mL. Internal standard (IS) was added to each sample of CS or QC to the final concentration of 9.6 ng/mL.

Stock solutions (100 *μ*g/mL) of IS propranolol were prepared by exactly weighing 0.6 mg and dissolving it with 6.0 mL CH_3_OH. IS working solutions (100 ng/mL) were obtained by diluting with CH_3_OH. Lenvatinib stock solution was stored at −70°C. IS working solution was prepared immediately before every experiment.

Plasma samples were prepared according to the method reported by Dubbelman et al. [5]. Drug-free control human plasma originated from healthy volunteers.

Plasma samples were subjected to protein precipitation. IS working solutions (30 *μ*L; 100 ng/mL) and 500 *μ*L acetonitrile were added to 250 *μ*L plasma samples. After shaking for 10 min at 1250 rpm on an automatic shaker at 25°C, the samples were centrifuged for 5 min at 20000 ×g. The supernatants were transferred to clean tubes and desiccated by N_2_ gas at 40°C. The dried samples were redissolved with 0.1% formic acid in 75% acetonitrile/water (v/v), vortexed for 30 s, and centrifuged at 20,000 ×g for 5 min. The resulting clear solutions were injected (5 *μ*L) into the HPLC column for further analyses.

### 2.6. Validation Procedures

The quantitative assay of lenvatinib in human sodium heparinized plasma was fully validated according to the Guideline on Bioanalytical Method Validation in Pharmaceutical Development in Japan [7] by testing accuracy, precision, linearity, range, selectivity, lower limit of quantification (LLOQ), recovery, and matrix effect.

## 3. Results and Discussion

### 3.1. Limit of Detection and Quantitation

**Figure 1:**
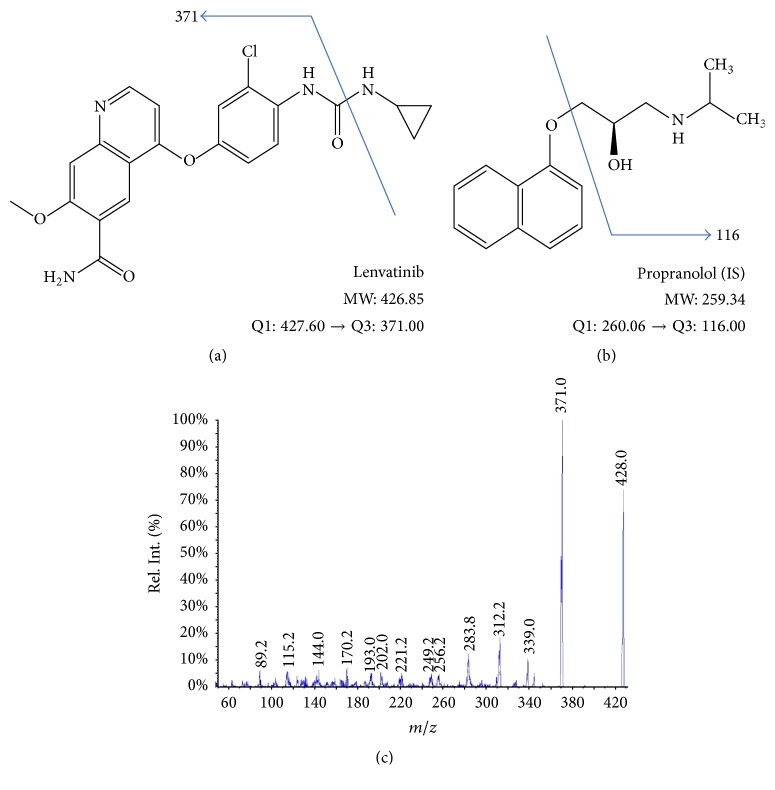
Chemical structures of lenvatinib and propranolol as an internal standard. Product ion mass spectra of lenvatinib. Chemical structure of lenvatinib (a) and propranolol (b) as an internal standard. (c) Product ion spectrum of lenvatinib at a precursor ion *m*/*z* 427.60.

**Figure 2:**
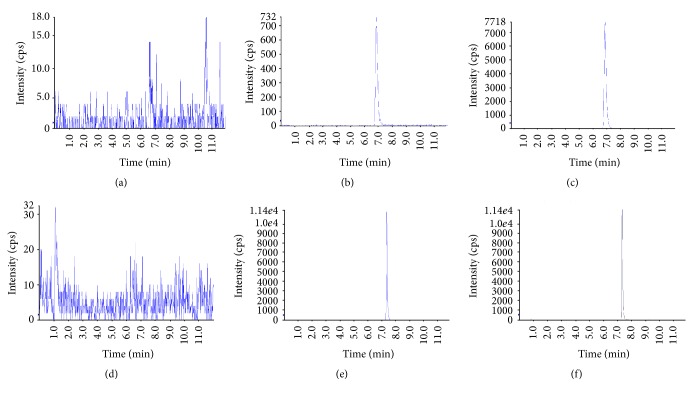
MRM chromatograms of the specimens. Representative MRM chromatograms of (b) lenvatinib (LOD; 0.96 ng/mL), (c) lenvatinib (LLOQ; 9.6 ng/mL), (e) propranolol as an internal standard, (a), (d) blank samples (plasma), and (f) zero sample (blank sample spiked with internal standard).

The chemical structures of lenvatinib and propranolol as an internal standard and product ion spectrum of lenvatinib at a precursor ion *m*/*z* 427.603 are shown in Figure 1. Retention times of lenvatinib and propranolol (IS) were approximately 6.8 min and 7.1 min, respectively (Figure 2). There were no interfering peaks (signal-to-noise ratio of >5) at these retention times in the multiple reaction monitoring (MRM) chromatograms. No carry-over peaks (0.1–1%) were detected in the subsequent chromatograms of plasma samples. The limits of detection (LOD) and quantitation (LOQ) were 0.96 ng/mL and 9.6 ng/mL, respectively.

### 3.2. Linearity

Propranolol was easily ionized in the positive-mode LC-MS/MS, and it had no ion suppression or enhancement effects on the analyte. Therefore, propranolol was considered appropriate as an internal standard.

The calibration plot was linear over the concentration range of 9.6–200 ng/ml (*n* = 6 for 6 different concentrations), which was the target concentration of pharmacokinetic (PK) study. The regression equation was *y* = 0.15*x* − 0.0103 (*x*-axis of the calibration curve is analyte concentration/internal standard concentration, and *y*-axis is analyte area/internal standard area.), where *R*^2^ value was 0.997. A weighing factor of 1/*x*^2^ on the calibration data was chosen since it resulted in smaller differences between the back-calculated and the nominal concentrations. At all concentrations, the deviations of the back-calculated concentrations were within 15% of the nominal concentrations with a CV below 15%.

If samples required dilution before analysis, then dilution integrity should be tested to confirm no impact on the measured concentration of the analyte. The dilution integrity was evaluated by 5 replicates per dilution factor after diluting a sample with blank matrix to bring the analyte concentration within the calibration range. QC > ULOQ (upper limit of quantification) samples were diluted 5 and 20 times in the human plasma. The precision and accuracy (RE%) of the method ranged from 4.1 to 15% and within ±1.9%, respectively.

### 3.3. Accuracy and Precision

The accuracy and precision of the assay were assessed by analyzing 6 nonzero calibration standard samples and 4 quality control samples (QC-LLOQ (quality control-lower limit of quantification), QC-low, QC-middle, and QC-high) in 5 (QC samples) or 6 (CS samples) times on 3 different occasions.

The intraassay precision (CV%) and accuracy (RE%) were 4.97–6.73% and within ±3.4%. Additionally, interassay precision (CV%) and accuracy (RE%) were 2.38–6.73% and ±8.3%, respectively. At all concentration levels, the accuracy and precision were within ±15%.

### 3.4. Specificity and Selectivity

The specificity and selectivity of assay were tested by analyzing blank samples (plasma containing neither analyte nor IS) and LLOQ samples in 6 individual batches.

None of the MRM chromatograms of the double blank samples showed peaks coeluting with an area of >20% of LLOQ. Additionally, no peaks of >5% of IS peak area were present in the plasma. The accuracy and precision of analyte LLOQ were −4.7–4.1% and 9.1–17.3%, respectively.

### 3.5. Total Recovery and Matrix Effect

To test the total recovery and matrix effect, 3 replicates of 3 analyte concentration levels (QC-low, QC-middle, and QC-high) and one IS concentration level (9.6 ng/mL) were investigated. The total recovery was determined by comparing the analyte response in a biological sample that was spiked with the analyte and processed with the analyte response in a biological blank sample that was processed and then spiked with the analyte. In the plasma, the recovery yields of analyte and IS were 70.4% (QC-low), 74.1% (QC-middle), 68.9% (QC-high), and 62.5% (IS) with standard deviation (SD) values of 1.8%, 1.9%, and 1.3%, respectively. The matrix effect ranged from +12.9 to +15.7%, which was within ±15%.

### 3.6. Carry-Over

Carry-over was investigated by analyzing a blank sample following the highest concentration calibration standard in 6 replicates. The response in the blank sample obtained after the highest concentration standard should not be greater than 20% of the analyte response of the LLOQ and also not greater than 5% of the response of internal standard. Carry-over was considered acceptable.

### 3.7. Stability Test

Lenvatinib stock solution was stored at −70°C. After one month, more than 95.7% of the drug could be recovered. The stock solutions were stable for at least one month.

The long-term stability of QC-low and QC-high concentration levels stored at −20°C for one month was assessed by calculating the recovery rate against the initial concentrations (*n* = 3). At this condition, the recovery rate of lenvatinib was >95%.

The stability after 3 freeze and thaw cycles was demonstrated by calculating the deviation from the initial concentration in 3 replicates. The recovery rates at the concentrations levels of QC-low and QC-high were 90.4% and 86.5%, respectively.

The short-term stability experiments at room temperature for 24 hrs were executed by using three replicates at the concentrations levels of QC-low and QC-high. The recovery rates were 98.2% (QC-low) and 98.6% (QC-high).

The autosampler stability was determined by storing the QC-low and QC-high concentration samples for 24 hrs in the autosampler (*n* = 3). The recovery rates at the concentrations levels of QC-low and QC-high were 92.0% and 95.6%, respectively.  Table 1 summarizes the results of various stability tests.

### 3.8. Overall Assessment

LC-MS/MS assay by using propranolol as an internal standard for the quantification of lenvatinib in human plasma has been developed and validated to meet the Guideline on Bioanalytical Method Validation in Pharmaceutical Development in Japan [7]. The results are in accordance with two previous studies by using ER-227326-00 as an internal standard presented by Dubbelman et al. [5] and Mano and Kusano [6].

In the future, we propose to conduct a PK study in patients with thyroid cancer receiving lenvatinib. Thyroid cancer patients are often accompanied by dysphagia with disease progression [8–10]. The patients with dysphagia have difficulty swallowing tablets or capsules; therefore, tablets or capsules are often crushed into powder or liquid suspension to be administered via an eternal tube [11–15]. However, bioavailability of the antineoplastic agent through different routes of administration (i.e., oral versus eternal tube) has not been clear. Therefore, we plan to investigate the equivalence of pharmaceutical formulations.

## 4. Conclusion

A liquid chromatography-tandem mass spectrometric assay for quantitative analysis of lenvatinib in human plasma was validated in this study. The calibration plot was linear over the concentration range of 9.6–200 ng/ml. At all concentration levels, the accuracy and precision were within ±15%. Collectively, this assay is applicable to conduct a PK study of thyroid cancer subjects with dysphagia.

## Figures and Tables

**Table 1:** Stability of lenvatinib in human plasma and stock solutions.

Condition	Matrix	Mean nominal conc.	Mean measured conc.	Recovery	CV
		ng/mL	ng/mL	%	%
−70mLc.d1 month	3.3 mM HCl/CH_3_OH	57800	55300	95.7	2.4
−2070HHC month	Plasma	9.6	9.2	95.8	1.6
−208aHHC month	Plasma	200.6	201.3	100.3	0.46
3 freeze and thaw cycles	Plasma	11.5	10.4	90.4	1.6
3 freeze and thaw cycles	Plasma	179.8	155.6	86.5	0.79
Room temperature for 24 hours	Plasma	11.5	11.3	98.2	2.6
Room temperature for 24 hours	Plasma	179.8	177.4	98.6	2.8
Autosampler	After treatment	10.1	9.3	92	7.3
Autosampler	After treatment	199.3	190.6	95.6	1.8
